# Effect of Cardiac Rehabilitation on Quality of Life, Depression and Anxiety in Asian Patients

**DOI:** 10.3390/ijerph15061095

**Published:** 2018-05-28

**Authors:** Carol C. Choo, Peter K. H. Chew, Shuet-Ming Lai, Shuenn-Chiang Soo, Cyrus S. Ho, Roger C. Ho, Raymond C. Wong

**Affiliations:** 1 College of Healthcare Sciences, James Cook University, Singapore 387380, Singapore; peter.chew@jcu.edu.au; 2 National University Heart Centre, National University Health System, Singapore 119007, Singapore; shuet_ming_lai@nuhs.edu.sg (S.-M.L.); raymond_cc_wong@nuhs.edu.sg (R.C.W.); 3 Department of Psychological Medicine, National University of Singapore, Singapore 119007, Singapore; shuenn_chiang_soo@nuhs.edu.sg (S.-C.S.); su_hui_ho@nuhs.edu.sg (C.S.H.); pcmrhcm@nus.edu.sg (R.C.H.)

**Keywords:** cardiac rehabilitation depression, anxiety, mental quality of life, physical quality of life

## Abstract

This study explored the effect of cardiac rehabilitation on quality of life, depression, and anxiety in Asian patients in Singapore. Out of the 194 patients who were recruited into the study, 139 patients (71.6%) completed both the pre- and post-cardiac rehabilitation questionnaires. Their ages ranged from 28 to 80 (M = 56.66, SD = 8.88), and 103 patients (74.1%) were males and 21 patients (15.1%) were females. As hypothesized, there was a statistically significant difference between the pre- and post-cardiac rehabilitation scores on the combined dependent variables, F (4, 135) = 34.84, *p* < 0.001; Wilks’ Lambda = 0.49; partial eta squared = 0.51. An inspection of the mean scores indicated that patients reported higher levels of physical and mental quality of life and lower levels of depression post-cardiac rehabilitation. The findings were discussed in regards to implications in cardiac rehabilitation in Singapore.

## 1. Introduction

There has been a shift in mental health services from an emphasis on treatment that is focused on reducing symptoms, to a more holistic approach, which considers quality of life and overall functioning [1]. Conversely, quality of life is an increasingly critical outcome of mental healthcare [2], and a lower quality of life has been associated with mental illness [3,4]. There is high prevalence of comorbidity between mental illness and physical illness [5,6], and one example is the high prevalence for depression in local Asian patients with myocardial infarction [7]. Both depression and anxiety were associated with worse clinical outcomes for cardiac patients [8]. However, evidence to support efficacy of treatment with psychotropic medication [9], and psychological therapy [10] ranged from weak to moderate, for patients with co-morbid conditions, namely myocardial infarction and depression. There is growing evidence in the literature highlighting the influence of psychological determinants in somatic diseases [11], suggesting that greater collaboration across allied health, medical, and psychiatric services is needed to implement multidisciplinary treatment, with a view to optimize clinical and holistic psychosocial outcomes for patients; however, specific multidisciplinary research in Asian cardiac patients is still lacking. 

Cardiac rehabilitation is a promising emerging multidisciplinary treatment [12] in the reduction of depression, anxiety [13], and enhancement of quality of life in patients with coronary heart disease [14,15]. A comprehensive cardiac rehabilitation program includes patient assessment, nutritional counseling, management of biopsychosocial, and metabolic cardiac risk factors (e.g., hypertension, lipids, diabetes), as well as physical activity counseling and psychosocial management [12]. In the psychosocial assessment, the interviewer uses standardized measurement tools to identify psychological distress in terms of depression and anxiety levels. Psychosocial interventions include group education and counseling, stress management skills training, and support in health-related lifestyle changes. Family members are encouraged to be included, with appropriate referrals to mental health specialists if relevant. Cardiac rehabilitation is a secondary prevention treatment modality that aims to reduce mortality and morbidity among patients with coronary heart disease, with objectives to improve symptoms, functional capacity, metabolic status, and depression, to ensure that cardiac patients achieve optimal physical, mental, and social conditions, so that they may resume as normal a place as possible in the community. 

Cardiovascular disease is the leading cause of death globally, despite a greater burden of disease, ethnocultural minorities in Western countries e.g., the United States and Canada were significantly less likely to access, adhere to, and complete cardiac rehabilitation, which could increase their risk to experience recurrent cardiac events and unnecessarily premature death [16]. A study done in Canada [17] found that although South Asian Canadians experienced cardiovascular disease at significantly higher rates, they were the least likely ethnocultural minority group to complete cardiac rehabilitation (43% as compared with 51% of white patients). The need to develop and deliver ethnoculturally sensitive cardiac rehabilitation services was highlighted. Adding to the concern is a comparative lack of operational cardiac rehabilitation programs in Asian countries, e.g., in China, there were two cardiac rehabilitation programs for every 100 million inhabitants, which is significantly lower than most countries, such as the United States, where there were 10 cardiac rehabilitation programs per 100 million inhabitants [18]. Recent Asian literature in China [19] and India [18] highlighted possible barriers that might affect successful implementation of cardiac rehabilitation programs in Asia [18,19]. Such issues are related to awareness and understanding of the benefits of cardiac rehabilitation among local health-care professionals, referrals to cardiac rehabilitation, limited presence of specialized professionals trained in cardiac rehabilitation, and a lack of awareness among patients on the benefits of cardiac rehabilitation [18,19]. There is also a lack of consistent and robust Asian literature to support the use of cardiac rehabilitation. Cardiac rehabilitation did not show convincing results in Asian samples in Korea [20], while other Asian studies only investigated physical [21] and physiological outcomes [22,23], such studies were done in Singapore [21], China [22], and Korea [23]. In a recent Asian study that was done in Malaysia [24], cardiac rehabilitation was shown to increase physical quality of life, but mental quality of life decreased. Although cardiac rehabilitation showed promising results in Western studies, research is much needed in the local Asian population, as it cannot be assumed that cardiac rehabilitation practices in Western countries is also relevant to the local Asian population, as implementation of effective cardiac rehabilitation needs to take into consideration the local cultural context [25], and account for both physical and mental quality of life. 

This aim of the study was to explore the effect of a local cardiac rehabilitation program on depression, anxiety, and physical and mental quality of life of Asian patients with myocardial infarction in Singapore.

## 2. Materials and Methods 

### Procedure

Ethics approval was obtained from the Domains-Specific Review Board of National University Hospital, a large teaching hospital in Singapore. The approval code was National Healthcare Group Domain Specific Board 2012/00927. 194 patients with myocardial infarction were recruited at the National University Hospital. Individuals who met the inclusion criteria below were approached and were briefed by hospital clinical staff regarding the objectives of the study. Patients who agreed to be part of the study and gave consent were given the surveys thereafter. After completion of the surveys, the patients were subsequently debriefed, and thanked for the time spent in this study. Eligibility criteria for the study included the following: (a) above the legal age of consent, i.e., aged 21 years and above; (b) able to understand and respond to questions in English and/or Mandarin; and, (c) absence of intellectual impairment. The inclusion criteria were cardiac patients with acute coronary disease or myocardial infarction who agreed to attend at least 10 cardiac rehabilitation sessions at the National University Hospital. Patients who were being treated for existing severe and debilitating psychiatric co-morbidities with active florid psychotic symptoms were excluded e.g., psychotic disorder, dementia, substance dependence or abuse. Patients who entered the cardiac rehabilitation program for reasons not related to myocardial infarction or acute coronary disease were excluded e.g., post mitral valve replacement, weight loss program for patients with multiple cardiac risk factors. Patients with other severe medical conditions that might affect rehabilitation e.g., chronic obstructive pulmonary disease, renal failure, arthritis, and cancer were excluded. Data collection was conducted before and after the cardiac rehabilitation program. 

The patients completed at least 10 sessions of the cardiac rehabilitation program that lasted about one month. Demographic data, namely age, gender, marital status, and educational level were obtained from medical records. Cardiovascular risk factors, e.g., body mass index and patients' subjective quality of life, self-efficacy, perceived social support, and coping style were investigated with the use of self-report questionnaires. 

The cardiac rehabilitation program was developed by the local team of multidisciplinary cardiac experts to meet the needs of local cardiac patients and their caregivers. The program is a comprehensive, multidisciplinary program, comprising three primary services, namely education, counseling, and prescribed exercise training, and was organised into four phases. Phases 1 and 2 were conducted in the hospital, while phases 3 and 4 were conducted in the community. In phase 1, information was provided on cardiovascular disease, associated risk factors, and treatment modalities, including medications, as well as lifestyle and home-care advice. In phase 2, exercise training was prescribed by the cardiac rehabilitation physician and nurse specialists where personalized exercise goals were set with the patients, based on age and physical conditioning. Counseling support was also provided to patients and caregivers e.g., in life coaching and enhancing their coping mechanisms. These sessions were conducted in groups of 12 patients. In phase 3, referrals were made to psychiatrists, if relevant, and were based on individual patient needs. Phase 4 consisted of exercise training in the community gymnasium with the cardiac physiotherapists’ support.

Out of the 194 patients who were recruited into the study, 139 patients (71.6%) completed both the pre- and post-cardiac rehabilitation questionnaires. Their ages ranged from 28 to 80 (M = 56.66, SD = 8.88), 103 patients (74.1%) were males, and 21 patients (15.1%) were females, and 15 patients (10.8%) did not indicate their gender. In addition, 10 patients (7.2%) completed primary education, 50 patients (36.0%) completed secondary education, 61 patients (43.9%) completed tertiary education, and 18 patients (12.9%) did not indicate their education level. The dropout rate of 28.4% is consistent with a similar study that was done in the United Kingdom [26], and is lower than a recent study done in Brazil [27]. 

## 3. Measures

Pre- and post-cardiac rehabilitation depressive and anxiety scores were measured using the Hospital Anxiety and Depression Scale, HADS [28], a 14-item scale measuring anxiety and depression symptoms among medical outpatients. It was brief, well validated with research evidence [28], and easy to administer. Seven items were related to anxiety and depression, respectively. Each item was scored from 0 to 3, with scores ranging from 0 to 21 for either anxiety or depression. The cut off score was 8 for either domain. 

Pre- and post-cardiac rehabilitation quality of life was evaluated using the Short Form 12, SF-12 [29]. The SF-12 assessed patients’ subjective quality of life. Eight domains of health, which included physical functioning, role limitations due to physical and emotional problems, bodily pain, general health, vitality, social functioning, and mental health were assessed across 12 items. Higher scores denoted better perceived quality of life. The overall score was obtained from the eight scales and two measures were elicited: the physical component summary, which accounted for the individual’s physical health; and, the mental component summary, which accounted for the individual’s mental health. High test/retest validity and good internal reliability were derived with Asian populations [30].

## 4. Results

The data were analysed using IBM SPSS Statistics 25. The data were subject to Little’s [31] Missing Completely at Random, MCAR test [29]. The percentages of missing data across the variables ranged from 17.3% to 43.2%. The MCAR test suggested that the data were missing completely at random, χ^2^ = 55.42, *df* = 48, *p* > 0.05. The current study used the expectation-maximization method to address the problem of missing data, which is an effective method, when considering the rates of missing data in our study [32].

The means and standard deviations of the dependent variables are presented in Table 1. A one-way within-subjects MANOVA was performed to investigate the effect of cardiac rehabilitation on physical and mental quality of life, anxiety, and depression. There was a statistically significant difference between the pre- and post-cardiac rehabilitation scores on the combined dependent variables, F (4, 135) = 34.84, *p* < 0.001; Wilks’ Lambda = 0.49; partial eta squared = 0.51. When the results for the dependent variables were considered separately, the only difference to reach statistical significance, using a Bonferroni adjusted alpha level of 0.0125 (i.e., 0.05/four analyses), was physical quality of life, F (1, 138) = 88.35, *p* < 0.001; partial eta squared = 0.39, mental QOL *F* (1, 138) = 23.64, *p* < 0.001; partial eta squared = 0.15, and depression, F (1, 138) = 41.63, *p* < 0.001; partial eta squared = 0.23. An inspection of the mean scores indicated that patients reported higher levels of physical and mental quality of life, and lower levels of depression in the post-cardiac rehabilitation scores. 

## 5. Discussion

This study explored the effect of cardiac rehabilitation in 139 Asian cardiac patients in Singapore, on physical quality of life, mental quality of life, anxiety, and depression. As hypothesized, patients who completed at least 10 sessions of cardiac rehabilitation reported higher levels of physical and mental quality of life and lower levels of depression post- cardiac rehabilitation. The finding that patients reported higher post-rehabilitation physical quality of life and lower depression is consistent with previous literature [13,15].

Most Asian studies investigated physical quality of life [23]. Our finding is consistent with a recent Asian study, which found an improvement in physical quality of life [24]. The finding that cardiac patients reported higher post-cardiac rehabilitation mental quality of life and lower depression significantly adds to recent Asian literature. Future research could further explore symptoms of depression, e.g., somatic symptoms of depression are frequent among Asian patients with depression and are associated with greater clinical severity [33], pain symptoms might also impact on severity of depression and quality of life [34] and make these clinical symptoms more resistant to treatment. Qualitative interviews could explore the underlying mechanisms and specific aspects of cardiac rehabilitation that impact on physical and mental quality of life and specific symptoms of depression. 

The results of the study need to be interpreted with caution. Limitations to the study included the following: A lack of a control group limited how the data could be interpreted. Future research could compare quality of life, depression, and anxiety between cardiac patients who went through cardiac rehabilitation with a wait-listed control group for better interpretation of data, using randomized controlled trial. Follow up studies at 6 to 12 month intervals could explore the sustained effect of cardiac rehabilitation over time. Other limitations of the study included the reliance on self-report. Future research could seek patients’ consent to interview family members for corroboratory and collateral information.

Another aspect of cardiac rehabilitation, which is related to non-adherence to the cardiac rehabilitation program, could be further investigated, in order to obtain information, which could be used to further refine and tailor the program to optimize adherence [35]. Future study could investigate the varied reasons that might account for non-adherence, e.g., individual or intrapersonal factors that might contribute to the patients’ inability to comprehend efficacy and contents of the cardiac rehabilitation program [36] or busy work schedules [37]. Such variables are important to consider, in tailoring a program to maximize patient motivation and adherence and could be explored in qualitative interviews with patients who dropped out of cardiac rehabilitation. Patients’ perspectives on suitable strategies to enhance adherence and motivation could be explored in future research e.g., customizing and simplifying learning and intervention regimes, actively identifying barriers to adherence and addressing them, ensuring that patient support structures are in place and improving self-efficacy. Future directions could also explore research and development in educational design, use of technology to assist education; psychological intervention strategies to support learning, motivation, self-efficacy and behavior change; and, ways to improve healthcare providers' engagement with patients [38].

## 6. Conclusions

In conclusion, the findings shed light on multidisciplinary interventions and cardiac rehabilitation for local Asian cardiac patients. As is consistent with previous literature, there is an indication for greater referrals to cardiac rehabilitation [39], and wider publicity and education to both patients and clinical staff on benefits of cardiac rehabilitation [36]. Provision of cardiac rehabilitation training could also be provided to healthcare professionals at private clinics, and polyclinics to promote greater implementation of cardiac rehabilitation, and to increase the geographical coverage and accessibility of cardiac rehabilitation [19]. There is indication for greater scope in multidisciplinary assessment, management, and monitoring of both physical and mental outcomes, with a routine tool e.g., PHQ-9 being used to screen and monitor depressive symptoms in cardiac patients, to inform appropriate referrals. The PHQ-9 had been used in Asian cardiac samples [22].

By using interventions that are substantiated by empirical findings from current research in the local population, the clinician could be taking a step forward in utilizing the scientist-practitioner model in their evidence-based practice. This study adds to the current literature on cardiac rehabilitation, especially for Asian patients in the region, and it draws further focus to the importance of multidisciplinary interventions to enhance both physical and mental wellbeing and the quality of life of cardiac patients.

## Figures and Tables

**Table 1:** Means and Standard Deviations of Physical and Mental Quality of Life [QOL], Anxiety, and Depression Scores Pre- and Post-Cardiac Rehabilitation [CR].

Variables	Pre-CR		Post-CR	
	M	SD	M	SD
Physical QOL	41.38	7.67	47.23	6.76
Mental QOL	49.95	7.89	52.48	5.90
Anxiety	4.46	3.50	3.96	2.75
Depression	3.95	2.73	2.51	2.32
